# Fucoidan-Mediated Biogenic Gold Nanoparticles from *Padina tetrastromatica*: In Vitro and In Silico Evaluation of Multifunctional Biological Activities

**DOI:** 10.3390/ph19070976

**Published:** 2026-06-23

**Authors:** Ahmed S. El Newehy, Mostafa E. Elshobary, Mona M. Ismail, Abdulelah S. Alrebaish, Adam A. Sulaiman, Dara Aldisi, Mahmoud M. A. Abulmeaty, Saly F. Gheda

**Affiliations:** 1 Department of Community Health Sciences, College of Applied Medical Sciences, King Saud University, Riyadh 11433, Saudi Arabia; daldisi@ksu.edu.sa (D.A.); mabulmeaty@ksu.edu.sa (M.M.A.A.); 2 Botany and Microbiology Department, Faculty of Science, Tanta University, Tanta 31527, Egypt; salygheda@yahoo.com; 3 National Institute of Oceanography and Fisheries (NIOF), Cairo 11516, Egypt; mona_es5@yahoo.com; 4 Department of Biomedical Technology, College of Applied Medical Sciences, King Saud University, Riyadh 11433, Saudi Arabia; aalrebaish@ksu.edu.sa; 5 Department of Environmental Health and Safety, King Fahd University of Petroleum and Minerals, Dhahran 31261, Saudi Arabia; adamahmed@kfupm.edu.sa

**Keywords:** fucoidan, gold nanoparticles, hepatocellular carcinoma, biogenic synthesis, molecular docking, cytotoxicity

## Abstract

**Purpose:** This study sought to extract and characterize fucoidan from brown seaweed *Padina tetrastromatica* for the synthesis of fucoidan–gold nanoparticles (F-AuNPs) and to assess their physicochemical properties, as well as their antioxidant, anti-inflammatory, and anticancer activities, alongside potential molecular interactions with specific cancer-related targets. **Methods:** The extracted fucoidan-rich fraction was characterized for its sulfate content. Citrate-stabilized plain gold nanoparticles (plain AuNPs) were prepared and characterized as non-fucoidan nanoparticle controls. Comprehensive physicochemical characterization, including UV–Vis spectroscopy, Fourier-transform infrared spectroscopy (FTIR), transmission electron microscopy (TEM), X-ray diffraction (XRD), dynamic light scattering (DLS), zeta-potential analysis, and thermogravimetric analysis (TGA), was performed on the resultant fucoidan-functionalized AuNPs (F-AuNPs). Biological activities were assessed using different techniques: antioxidant potential (Ferric Reducing Antioxidant Power (FRAP) and 2,2-diphenyl-1-picrylhydrazyl (DPPH) assays), anti-inflammatory effects (NO inhibition in macrophages), and anticancer efficacy against HepG2 cells (MTT and flow cytometry). Potential molecular targets relevant to these activities were further explored in silico using molecular docking against key cancer-related proteins, providing hypotheses for future experimental validation. **Results:** The fucoidan-rich fraction showed a sulfate content of 10.08%. Strong antioxidant activity was observed, especially in FRAP (11.20 ± 0.29 mg TE g^−1^ DW). F-AuNPs exhibited enhanced cytotoxicity against HepG2 cells (IC_50_ 138.1 µg mL^−1^) compared to plain AuNPs (IC_50_ 271.2 µg mL^−1^) and the fucoidan-rich fraction (IC_50_ 390.2 µg mL^−1^), inducing G1 phase arrest. In addition, F-AuNPs reduced nitric oxide production in LPS-stimulated RAW 264.7 macrophages, reaching 21.42 ± 1.29% inhibition at 100 µg mL^−1^. As an exploratory, hypothesis-generating step, an in silico target-prioritization screen identified HPSE and MMP-2 as the highest-scoring candidate proteins, proposed solely as targets for future experimental validation. **Conclusions:** F-AuNPs represent a promising multifunctional nanoplatform with antioxidant, anti-inflammatory, and antiproliferative activities. The integration of in vitro biological evaluation with in silico target prediction supports the potential biomedical relevance of F-AuNPs and generates testable hypotheses regarding their molecular targets, which require experimental validation.

## 1. Introduction

The epidemiological profile of primary liver cancer—dominated primarily by Hepatocellular Carcinoma (HCC), which constitutes nearly 80% to 90% of all cases globally—is routinely tracked by the World Health Organization (WHO) and its specialized oncology agency, the International Agency for Research on Cancer (IARC) [1]. HCC is closely linked to chronic liver inflammation, with 80–90% of cases associated with hepatitis B or C infection [2,3]. Hepatocytes are major producers and targets of reactive oxygen species (ROS) at the cellular level. Persistent oxidative stress accelerates the transition from chronic hepatitis to HCC by promoting inflammation, genomic instability, and progressive tissue damage [4,5].

These observations position ROS not only as pathological by-products but also as actionable therapeutic targets in liver cancer. Current therapeutic options for HCC, including surgical resection, liver transplantation, transarterial chemoembolization (TACE), radiofrequency ablation, and systemic agents such as sorafenib, lenvatinib, and immune checkpoint inhibitors, have improved patient outcomes but remain limited by several constraints [2,3]. Surgical and locoregional interventions are restricted by tumor stage, liver function, and donor availability, while systemic therapies are associated with adverse effects, including hand-foot skin reaction, hypertension, hepatic decompensation, gastrointestinal toxicity, and the eventual development of drug resistance [2,3].

Furthermore, the limited tumor selectivity of conventional chemotherapy contributes to off-target toxicity in healthy hepatocytes, underscoring the need for selective, biocompatible, and multifunctional therapeutic strategies that combine antioxidant, anti-inflammatory, and antiproliferative properties within a single platform. Alongside advancements in oncology, nanomedicine has transformed cancer therapy by enabling precise control over material properties at the nanoscale [6,7,8,9]. Among nanomaterials, AuNPs are particularly attractive for their optical tunability, chemical inertness, and excellent biocompatibility, facilitating applications in imaging, drug delivery, and anticancer therapy [10,11,12]. However, many conventional synthesis methods rely on hazardous chemicals and energy-intensive processes, raising concerns regarding sustainability and translational safety [13]. To mitigate these limitations, green synthesis methods utilizing biological systems have gained significant interest. Natural biomolecules can act as reducing and stabilizing agents, enabling environmentally sustainable nanoparticle synthesis while simultaneously incorporating biologically active surface chemistries [14,15]. Recent reports have demonstrated successful plant-mediated synthesis of gold and silver nanoparticles using various botanical sources, including leaves, fruits, peels, and seeds, which provide bioactive phytochemicals such as flavonoids, terpenoids, and phenolic acids capable of reducing metal precursors and stabilizing the resulting nanoparticles [16,17]. These plant-derived systems have shown promising antioxidant, antimicrobial, and anticancer activities, supporting the relevance of biologically derived reducing agents in nanomedicine [16,17].

Beyond terrestrial plants, marine algae represent an underexploited yet highly rich source of multifunctional biomolecules, including polysaccharides, phenolics, and proteins, many of which exhibit intrinsic pharmacological activities [18,19,20]. Fucoidan, a sulfated polysaccharide primarily extracted from brown marine algae, has gained substantial attention among algal-derived biomolecules due to its structural diversity and broad spectrum of bioactivities, including antioxidant, anti-inflammatory, and anticancer activity [21]. The biological efficacy of fucoidan is strongly influenced by algal species, sulfation patterns, and extraction conditions, suggesting that fucoidan from different sources should not be considered functionally identical. *P. tetrastromatica*, a brown alga of the Dictyotaceae family, is recognized for its content of fucoidan and other phytochemicals, including flavonoids, tannins, and phenolic compounds [22]. Although previous studies have explored *Padina*-derived algal extracts or fucoidan-based systems for nanoparticle synthesis and anticancer applications [23,24], several knowledge gaps remain. First, most prior reports have employed crude algal extracts rather than purified, sulfate-quantified fucoidan fractions, limiting structure–activity interpretation. Second, biological evaluations have typically focused on a single activity, such as antibacterial or antiproliferative activity, with limited integration of antioxidant, anti-inflammatory, and anticancer assessments within the same nanoplatform. Third, cytocompatibility toward normal hepatocytes and integrated in silico target prediction have rarely been addressed in parallel.

The present study was designed to address these gaps by employing a chemically characterized *P. tetrastromatica* fucoidan-rich fraction with quantified sulfate content as both a reducing/stabilizing agent and a bioactive surface corona for AuNP synthesis. The resulting F-AuNPs were subjected to comprehensive physicochemical characterization, multi-axis bioactivity evaluation against HepG2 cancer cells and normal BNL hepatocytes, and in silico docking against extracellular matrix- and immune-signaling-related targets. To distinguish the contribution of the gold core from that of the fucoidan corona, plain AuNPs (plain AuNPs) were synthesized in parallel as a non-fucoidan nanoparticle reference.

## 2. Results

### 2.1. Extraction of Fucoidan

**Figure 1:**
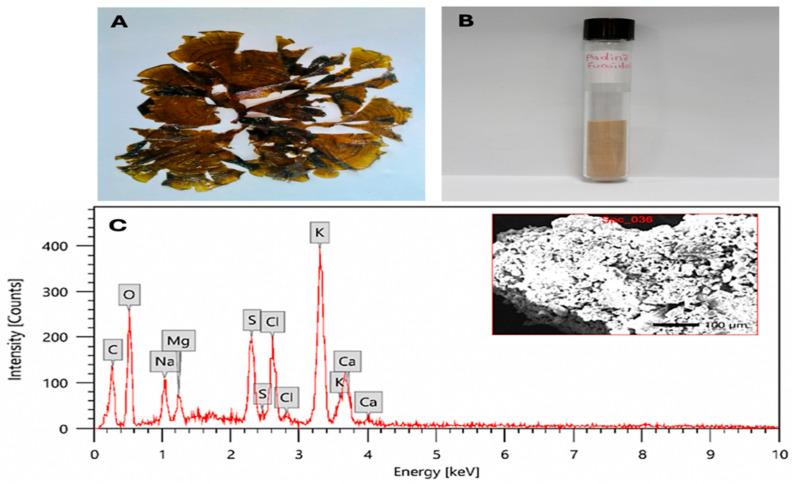
Visual and elemental characterization of the fucoidan-rich fraction from *Padina tetrastromatica*. (**A**) Cleaned *P. tetrastromatica* thalli before extraction. (**B**) Dried fucoidan-rich fraction after extraction and freeze-drying. (**C**) EDX spectrum showing elemental composition, including sulfur signals consistent with sulfated polysaccharides.

Representative photographs of the cleaned *Padina tetrastromatica* thalli before extraction and the obtained lyophilized fucoidan-rich fraction after extraction are presented in Figure 1A,B. The collected brown algal material showed the characteristic fan-shaped thallus morphology of *Padina*, while the recovered fucoidan-rich fraction appeared as a dried powder following ethanol precipitation, repeated washing, and freeze-drying. The fucoidan extraction yield from *P. tetrastromatica* was 178.3 mg g^−1^ dry weight (DW), corresponding to approximately 17.83% of the dry biomass.

### 2.2. Characterization of P. tetrastromatica Fucoidan

#### 2.2.1. Sulfate Content of *P. tetrastromatica* Fucoidan

The sulfate content of the extracted fucoidan-rich fraction was determined by the BaCl_2_–gelatin turbidimetric assay using sodium sulfate (Na_2_SO_4_) as the reference standard. The calibration curve was linear over the range 0–80 µg mL^−1^ (y = 0.0094x − 0.0038; R2 = 0.9991). The mean absorbance of the fucoidan hydrolysate at 360 nm was 0.323 ± 0.003 (n = 3), corresponding to a sulfate concentration of 25.19 µg mL^−1^ in the diluted sample. Following correction for the dilution factor, the sulfate content of the fucoidan-rich sulfated polysaccharide was 10.08% (*w*/*w*) (Supplementary Figure S1).

#### 2.2.2. EDX Analysis of *P. tetrastromatica* Fucoidan

The elemental composition of fucoidan extracted from *Padina tetrastromatica* was analyzed using energy-dispersive X-ray spectroscopy (EDX), as reported in Figure 1. The EDX spectrum demonstrated that fucoidan is primarily composed of oxygen (O) and carbon (C), with weight percentages of 37.30 wt% (42.31 at%) and 24.85 wt% (37.56 at%), respectively. This elemental distribution is consistent with the polysaccharide backbone of fucoidan. Notably, a relatively high sulfur (S) content (~16.25 wt%) was detected, which is a defining feature of sulfated polysaccharides and provides strong evidence for the presence of sulfate ester groups within the fucoidan structure. The elevated sulfur signal confirms the successful extraction of sulfated fucoidan from the brown seaweed. In addition, trace amounts of Na, Mg, K, Ca, and Cl were observed. The repeated labels observed for some elements in the EDX spectrum correspond to different characteristic X-ray emission lines, mainly Kα and Kβ transitions, rather than separate elemental species. This is a normal feature of EDX spectra and does not indicate duplication or the presence of different forms of the same element.

### 2.3. Characterization of P. tetrastromatica F-AuNPs

#### 2.3.1. Visual Confirmation of F-AuNP Formation

**Figure 2:**
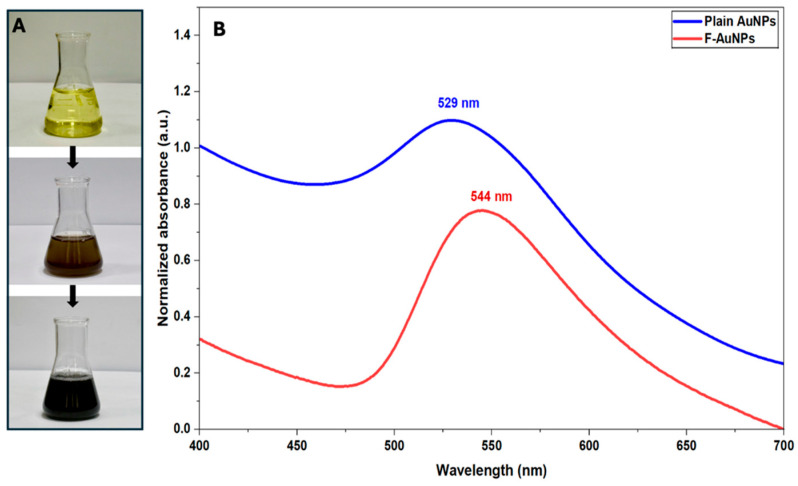
Synthesis and UV–Vis characterization of plain AuNPs and *P. tetrastromatica* F-AuNPs. (**A**) Visual color change observed during F-AuNP formation. (**B**) UV–Vis spectra of plain AuNPs and F-AuNPs showing SPR peaks at 529 and 544 nm, respectively.

The gradual transition in solution color from pale yellow to dark brown as shown in Figure 2A indicates the reduction of Au^3+^ ions and the formation of fucoidan-functionalized gold nanoparticles via surface plasmon resonance.

#### 2.3.2. UV–Vis Analysis of Plain AuNPs and *P. tetrastromatica* F-AuNPs

The UV–Vis absorption spectra of plain AuNPs and *P. tetrastromatica* F-AuNPs are shown in Figure 2B. Plain AuNPs exhibited a characteristic localized surface plasmon resonance (LSPR) band at approximately 529 nm, confirming the formation of metallic AuNPs. In comparison, F-AuNPs displayed a distinct SPR band at approximately 544 nm. The red shift from 529 to 544 nm may be attributed to fucoidan-mediated surface functionalization, changes in the local dielectric environment surrounding the gold core, and an increase in hydrodynamic size after biomolecular capping. The presence of a single dominant SPR band for F-AuNPs is consistent with predominantly spherical to quasi-spherical nanoparticles, as supported by TEM analysis, while the absence of a broad secondary band at longer wavelengths suggests no severe aggregation under the tested conditions.

#### 2.3.3. FTIR Analysis of the Fucoidan-Rich Fraction and *P. tetrastromatica* F-AuNPs

**Figure 3:**
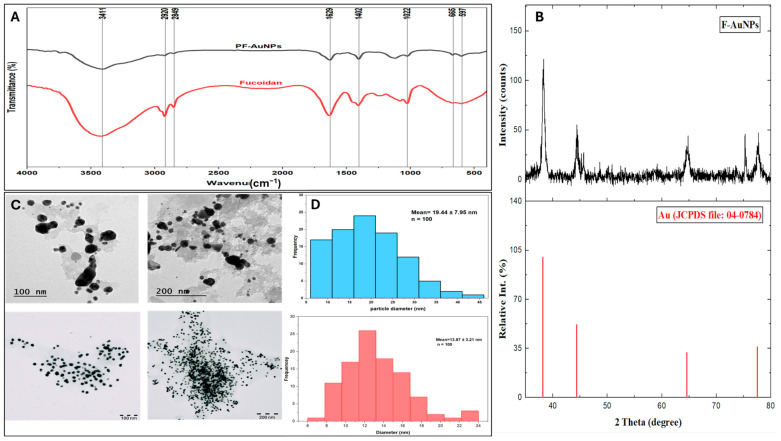
Structural and morphological characterization of the fucoidan-rich fraction, plain AuNPs, and *P. tetrastromatica* F-AuNPs. (**A**) FTIR spectra of the fucoidan-rich fraction and F-AuNPs showing functional groups involved in nanoparticle stabilization. (**B**) XRD pattern of F-AuNPs compared with the reference face-centered cubic gold pattern (JCPDS no. 04-0784). (**C**) Representative TEM micrographs and TEM-derived particle-size distribution of *P. tetrastromatica* F-AuNPs. (**D**) Representative TEM micrographs and TEM-derived particle-size distribution of plain AuNPs. Particle-size distributions were generated from 100 individually measured nanoparticles using ImageJ/Fiji (2.14.0).

FTIR transmittance spectrum of fucoidan showed a broad absorption band observed at approximately 3411 cm^−1^, which is assigned to O–H stretching vibrations, and its broad nature is commonly attributed to hydrogen bonding as reported in Figure 3A. The C–H stretching region appears as weak bands at around 2922 and 2850 cm^−1^, indicating a low contribution of aliphatic organic groups. An absorption band detected near 1620 cm^−1^ is commonly associated with stretching vibrations of oxygen-containing functional groups, while a band around 1406 cm^−1^ corresponds to C–H bending vibrations. Several bands were observed in the fingerprint region (≈400–1800 cm^−1^), with prominent peaks at 1156 and 1022 cm^−1^, which are characteristic of C–O and C–O–C stretching vibrations of polysaccharide structures. Additional weak bands were detected at lower wavenumbers, including 838 cm^−1^, which indicated C–O–S bending and sugar ring skeletal vibrations, further confirming the polysaccharide nature of the fucoidan.

The FTIR transmittance spectrum of *P. tetrastromatica* F-AuNPs shows a stronger and broader absorption band centered at approximately 3455 cm^−1^, corresponding to O–H stretching vibrations (Figure 3A). Weak bands in the region of 2926 cm^−1^ and 2854 cm^−1^ are attributed to C–H stretching vibrations. A noticeable absorption band appears at around 1635 cm^−1^, which is commonly associated with stretching vibrations of oxygen-containing functional groups, along with a band near 1400 cm^−1^. In the region of 1000 cm^−1^–1300 cm^−1^, a prominent peak at approximately 1016 cm^−1^ was observed, corresponding to C–O stretching vibrations. A band at approximately 684 cm^−1^ was also detected within the fingerprint region.

#### 2.3.4. X-Ray Diffraction Analysis of F-AuNPs

Figure 3B illustrates how X-ray diffraction (XRD) was used to examine the crystalline structure of the F-AuNPs. The (111), (200), (220), and (311) crystallographic planes of face-centered cubic (fcc) gold are represented by the diffraction pattern’s distinctive Bragg reflections at 2θ values of about 38.2°, 44.4°, 64.6°, and 77.5°. The effective development of crystalline Au nanoparticles is confirmed by these reflections, which aligned well with the conventional diffraction data for metallic gold (JCPDS file no. 04-0784). The exceptional phase purity of the produced nanoparticles was demonstrated by the absence of any extra diffraction peaks associated with contaminants or secondary phases. The relatively broadened diffraction peaks further suggest the nanoscale dimension of the Au crystallites, while the absence of fucoidan-related peaks reflects their amorphous nature and surface coating rather than incorporation into the gold crystal lattice.

#### 2.3.5. TEM Analysis of Plain AuNPs and *P. tetrastromatica* F-AuNPs

Transmission electron microscopy (TEM) analysis showed that both plain AuNPs and F-AuNPs were predominantly spherical to quasi-spherical and generally dispersed on the TEM grid (Figure 3C,D). The TEM-derived particle-size distributions were generated by measuring individual nanoparticles using ImageJ/Fiji (2.14.0), and the corresponding raw diameter measurements are provided in Table S1. Plain AuNPs exhibited a core diameter range of 7.07–23.54 nm, with a mean diameter of 13.46 ± 3.28 nm, whereas F-AuNPs showed a broader size range of 6.36–42.68 nm, with a mean diameter of 19.44 ± 7.95 nm. These values represent dry metallic core diameters and are therefore distinct from the DLS-derived hydrodynamic diameters measured in aqueous suspension.

#### 2.3.6. Zeta-Potential and Dynamic Light Scattering (DLS) Analysis of Plain AuNPs and *P. tetrastromatica* F-AuNPs

**Figure 4:**
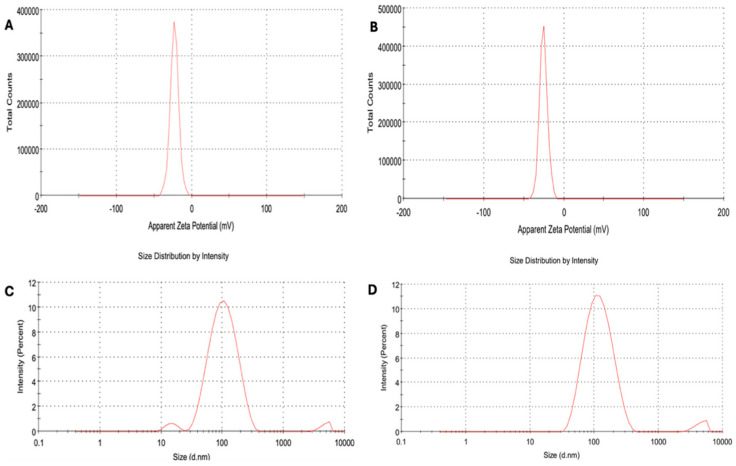
Comparative zeta-potential and hydrodynamic size characterization of plain AuNPs and *Padina tetrastromatica*-derived fucoidan-functionalized gold nanoparticles (F-AuNPs). (**A**,**B**) Zeta-potential distributions of plain AuNPs and F-AuNPs, respectively. (**C**,**D**) DLS intensity-based hydrodynamic size-distribution profiles of plain AuNPs and F-AuNPs, respectively.

Zeta-potential and DLS analyses were performed to compare plain AuNPs and *P. tetrastromatica* F-AuNPs (Figure 4). Plain AuNPs showed a negative zeta potential of −22.9 ± 5.75 mV, with a conductivity of 1.84 mS cm^−1^, whereas F-AuNPs exhibited a slightly more negative zeta potential of −25.4 ± 4.99 mV and a conductivity of 2.08 mS cm^−1^ (Figure 4A,B). DLS analysis showed that plain AuNPs had a Z-average hydrodynamic diameter of 85.72 nm with a PdI of 0.294, while F-AuNPs showed a larger Z-average hydrodynamic diameter of 99.21 nm with a PdI of 0.317 (Figure 4C,D). The increased hydrodynamic size and slightly more negative zeta potential of F-AuNPs compared with plain AuNPs support fucoidan-mediated surface functionalization and the formation of a hydrated polysaccharide corona around the gold core.

#### 2.3.7. Thermogravimetric Analysis of Plain AuNPs and *P. tetrastromatica* F-AuNPs

**Figure 5:**
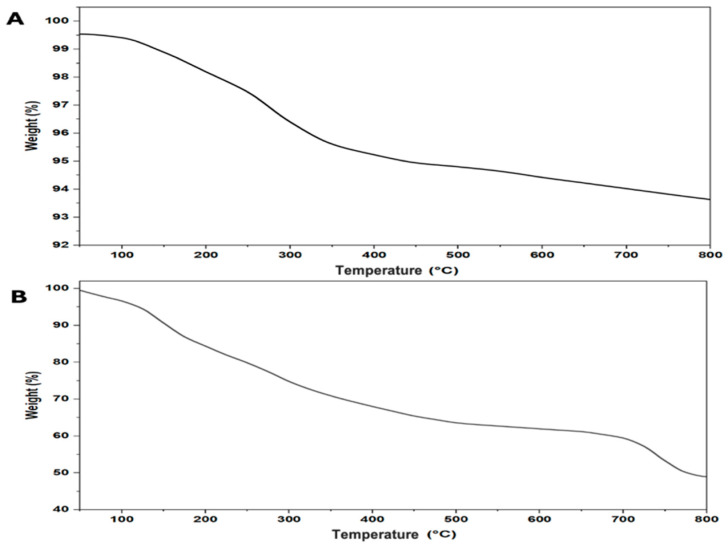
Thermogravimetric analysis (TGA) profiles of (**A**) plain AuNPs and (**B**) *P. tetrastromatica* fucoidan-coated AuNPs (F-AuNPs).

Thermogravimetric analysis (TGA) was performed to compare the thermal behavior of plain AuNPs and *P. tetrastromatica* fucoidan-functionalized AuNPs (F-AuNPs), and to assess the contribution of the organic surface coating (Figure 5A,B). Plain AuNPs showed only limited mass loss across the tested temperature range, retaining approximately 93–94% of their initial mass at 800 °C (Figure 5A). This minor weight reduction is consistent with the removal of adsorbed moisture and the thermal decomposition of low-abundance citrate species associated with the AuNP surface.

In contrast, F-AuNPs exhibited a more pronounced multi-step weight-loss profile, with a residual mass of approximately 48–50% at 800 °C (Figure 5B). The initial weight loss below approximately 150 °C can be attributed to the evaporation of physically adsorbed water and residual volatile components. The subsequent mass loss between approximately 150 and 350 °C is associated with the degradation of labile organic groups and low-molecular-weight fractions within the fucoidan-rich surface layer. Further gradual decomposition at higher temperatures reflects thermal degradation and carbonization of polysaccharide-associated organic components. Compared with plain AuNPs, the markedly greater mass loss observed for F-AuNPs supports the presence of a fucoidan-rich organic coating on the nanoparticle surface.

### 2.4. Biological Activities

#### 2.4.1. Antioxidant Activity of *P. tetrastromatica* F-AuNPs

The antioxidant activity of *P. tetrastromatica* F-AuNPs was evaluated using the FRAP and DPPH assays. The Trolox standard calibration curves used for quantification are presented in Supplementary Figures S2 and S3. Based on these calibration curves, the antioxidant capacity of the synthesized nanoparticles was determined and expressed as Trolox equivalents. The FRAP assay showed the highest antioxidant activity, yielding a value of 11.20 ± 0.29 mg TE g^−1^ DW. In contrast, the DPPH assay exhibited a lower radical scavenging capacity, with a value of 6.27 ± 0.64 mg TE g^−1^ DW. These results demonstrate that the synthesized F-AuNPs possess measurable antioxidant activity, with higher reducing power observed in the FRAP assay compared with the DPPH radical scavenging assay.

#### 2.4.2. MTT Assay for HepG2 Cells

**Figure 6:**
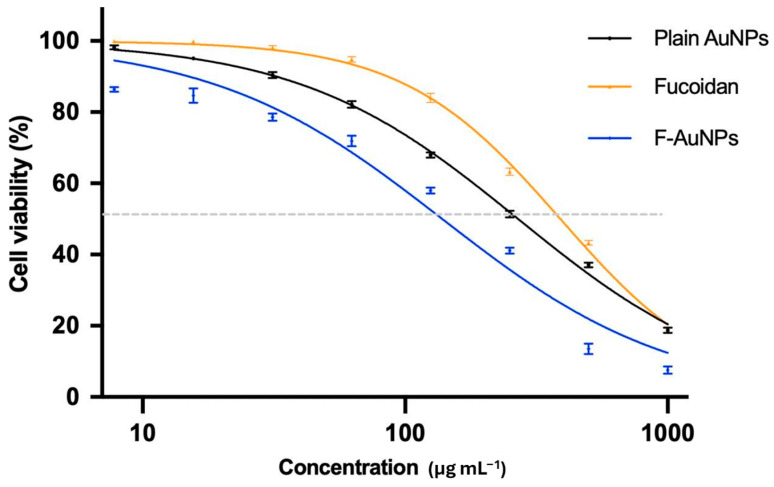
Dose-dependent cytotoxic effects of plain AuNPs, the fucoidan-rich fraction, and F-AuNPs against HepG2 cells assessed by MTT assay. Cell viability (%) was plotted against concentration (µg mL^−1^) using a log_10_-scaled *X*-axis. Data are presented as mean ± SD (n = 3). The dashed horizontal line indicates 50% cell viability for IC_50_ estimation.

Exposure of HepG2 cells to the fucoidan-rich fraction, plain AuNPs, and *P. tetrastromatica*-derived F-AuNPs resulted in a concentration-dependent decrease in cell viability. The IC_50_ values calculated using nonlinear dose–response fitting were 138.1, 271.2, and 390.2 µg mL^−1^ for F-AuNPs, plain AuNPs, and the fucoidan-rich fraction, respectively (Figure 6).

#### 2.4.3. Cytotoxicity Assessment of Normal Cells (BNL)

**Figure 7:**
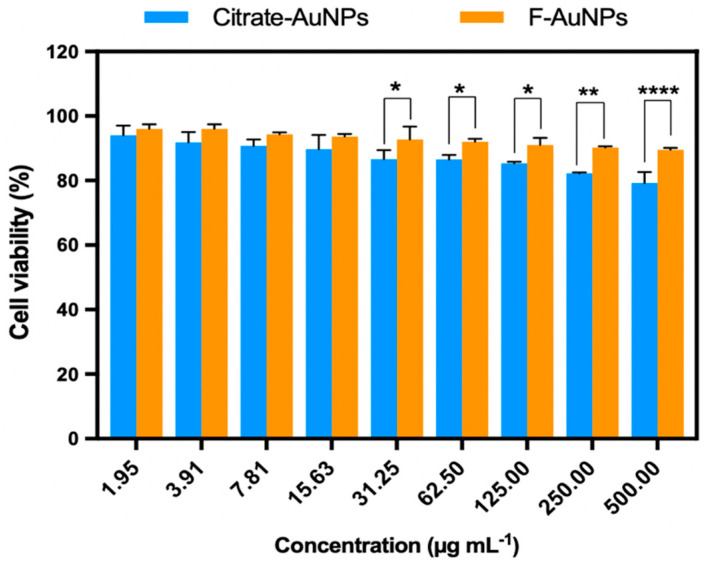
Cell viability of normal murine liver BNL cells following exposure to plain AuNPs and F-AuNPs. Cells were treated with increasing concentrations of plain AuNPs and F-AuNPs, and viability was expressed as mean ± SD (n = 3). Statistical comparisons between plain AuNPs and F-AuNPs at each concentration were performed using two-way ANOVA followed by Šídák’s multiple-comparison test. F-AuNPs maintained significantly higher cell viability than plain AuNPs at 31.25, 62.50, 125, 250, and 500 µg mL^−1^. * *p* < 0.05, ** *p* < 0.01, **** *p* < 0.0001.

The cytocompatibility of plain AuNPs and *P. tetrastromatica*-derived F-AuNPs was evaluated in normal murine liver BNL cells over the tested concentration range. Both nanoparticle formulations maintained relatively high cell viability, indicating limited toxicity toward normal liver cells under the applied conditions. However, F-AuNPs consistently preserved higher viability than plain AuNPs, with statistically significant differences observed at 31.25, 62.50, 125, 250, and 500 µg mL^−1^. These findings suggest that fucoidan coating improved the cytocompatibility profile of AuNPs compared with plain AuNPs (Figure 7).

#### 2.4.4. Flow Cytometric Evaluation of the Antiproliferative Effect of *P. tetrastromatica* F-AuNPs

**Figure 8:**
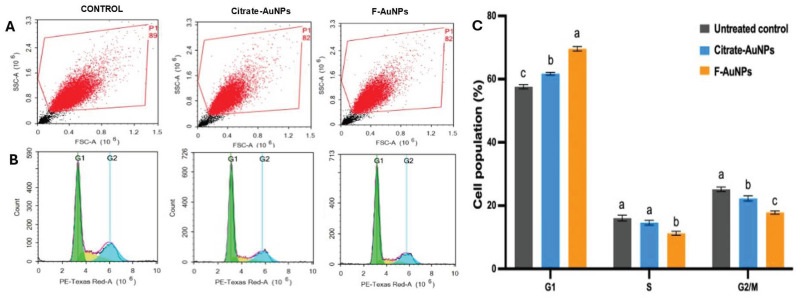
Flow cytometric cell-cycle analysis of HepG2 cells following treatment with plain AuNPs and F-AuNPs. (**A**) Representative FSC-A/SSC-A gating plots showing the selected P1 cell population used to exclude debris and non-representative events. (**B**) Representative DNA-content histograms showing cell-cycle phase distribution. HepG2 cells were treated with plain AuNPs and *P. tetrastromatica*-derived F-AuNPs at their respective IC_50_ concentrations, 271.2 and 138.1 µg mL^−1^, respectively. (**C**) Quantitative analysis of G1, S, and G2/M phase distribution. Data are presented as mean ± SD from three replicates. Green region: Represents the G1 phase, typically indicating cells with a single set of DNA (2N); Yellow region: Likely corresponds to the S phase, where DNA synthesis occurs (between 2N and 4N content); Blue region: Represents the G2/M phase, where cells have duplicated DNA content (4N). Statistical comparisons were performed among treatments within each cell-cycle phase using two-way ANOVA followed by multiple-comparison testing. Different letters indicate significant differences within the same phase at *p* < 0.05.

Flow cytometric cell-cycle analysis was performed to compare the effects of *P. tetrastromatica*-derived F-AuNPs and plain AuNPs on HepG2 cells (Figure 8). Cells were treated with plain AuNPs and F-AuNPs at their respective IC_50_ concentrations, 271.2 and 138.1 µg mL^−1^, respectively. Therefore, this comparison was based on each treatment’s IC_50_ concentration and was not an equal-dose comparison. The FSC-A/SSC-A scatter plots showed no major shift in the main cell population after nanoparticle treatment, suggesting no marked change in gross cell size or granularity. These plots were used mainly to define the P1 gate and exclude debris. Cell-cycle changes were then evaluated using PI-DNA content histograms.

The untreated control cells showed 57.61 ± 0.65% of cells in the G1 phase, 16.05 ± 0.91% in the S phase, and 25.17 ± 0.69% in the G2/M phase. Treatment with plain AuNPs induced a moderate redistribution of the cell-cycle profile, increasing the G1 population to 61.72 ± 0.42% and reducing the S and G2/M populations to 14.61 ± 0.74% and 22.27 ± 0.81%, respectively. In comparison, F-AuNPs produced a more pronounced effect, with a marked increase in the G1 population to 69.61 ± 0.73%, accompanied by reductions in the S phase to 11.24 ± 0.63% and the G2/M phase to 17.84 ± 0.55%.

Statistical analysis confirmed a significant treatment-dependent redistribution of HepG2 cells across the cell-cycle phases. F-AuNPs induced significantly higher G1-phase accumulation than both untreated control and plain AuNPs, while also producing significantly greater reductions in S and G2/M populations. These findings indicate that although plain AuNPs exerted a measurable cell-cycle effect, *P. tetrastromatica*-derived F-AuNPs displayed a stronger antiproliferative effect, consistent with enhanced G1-phase arrest and suppression of cell-cycle progression in HepG2 cells.

#### 2.4.5. Anti-Inflammatory Activity and Cytocompatibility in RAW 264.7 Macrophages

**Figure 9:**
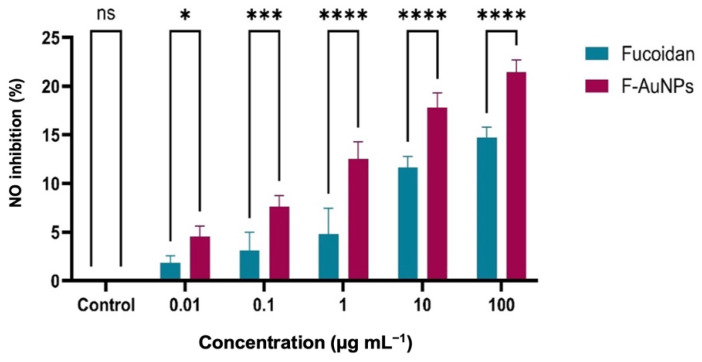
Inhibition of LPS-induced nitric oxide production by the fucoidan-rich fraction and F-AuNPs in RAW 264.7 macrophages. Data are presented as mean ± SD (n = 3). Statistical significance relative to the control is indicated (* *p* < 0.05, *** *p* < 0.01, **** *p* < 0.0001).

As shown in Figure 9, fucoidan and F-AuNPs derived from *P. tetrastromatica* exhibited a clear concentration-dependent inhibitory effect on lipopolysaccharide (LPS)-induced NO production in RAW 264.7 macrophages. Exposure to increasing concentrations of fucoidan and F-AuNPs (0.01–100 µg mL^−1^) resulted in a gradual and consistent reduction in NO levels compared with the LPS-treated control group. At lower concentrations, F-AuNPs produced a modest inhibitory effect, with NO inhibition reaching 4.55 ± 1.08% at 0.01 µg mL^−1^ while the NO inhibition of fucoidan reaching 1.83 ± 2.03% at 0.01 µg mL^−1^. This effect became progressively more pronounced with increasing concentrations, indicating a dose-responsive trend. At intermediate concentrations, a noticeable suppression of NO production was observed, while the highest concentration tested (100 µg mL^−1^) resulted in a maximum inhibition of 21.42 ± 1.29%, and 14.73 ± 1.04% for fucoidan. Statistical analysis revealed that NO inhibition at higher concentrations was highly significant relative to the control (*p* < 0.01–0.0001). The progressive decline in NO production across the tested concentration range demonstrates that F-AuNPs effectively attenuate inflammatory signaling in activated macrophages. This dose-dependent behavior suggests that the anti-inflammatory activity of F-AuNPs is closely associated with their concentration and supports their potential role as modulators of macrophage-mediated inflammatory responses.

#### 2.4.6. In Silico Target Prediction by Molecular Docking

A comparative in silico docking screen was performed to evaluate the predicted docking profiles of F-AuNPs and plain AuNPs against selected protein models related to extracellular-matrix remodeling, angiogenesis-associated signaling, and immune regulation. This analysis was included only as an exploratory computational comparison and was not intended to define a mechanism of action or to explain the biological activity observed in HepG2 cells.

**Figure 10:**
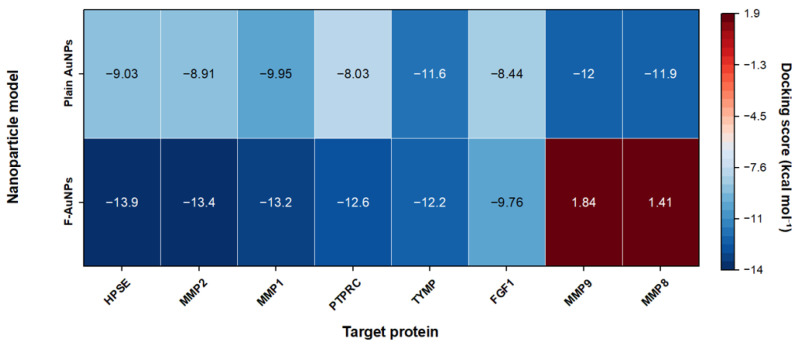
Heat map of docking ΔG (kcal mol^−1^) binding energy of F-AuNPs and plain AuNPs with protein receptors.

The docking heatmap showed that the predicted scores varied according to both the protein model and the nanoparticle model (Figure 10). F-AuNPs showed the most negative predicted docking scores with HPSE (−13.87 kcal mol^−1^), MMP2 (−13.42 kcal mol^−1^), MMP1 (−13.15 kcal mol^−1^), PTPRC (−12.60 kcal mol^−1^), and TYMP (−12.15 kcal mol^−1^). Compared with plain AuNPs, F-AuNPs showed more negative predicted scores for HPSE, MMP2, MMP1, PTPRC, TYMP, and FGF1 (Table S2).

The comparative pattern was not uniform across all screened protein models. For MMP9 and MMP8, F-AuNPs produced unfavorable positive docking scores of 1.84 and 1.41 kcal mol^−1^, respectively, whereas plain AuNPs showed more negative predicted scores for these two protein models. Therefore, the docking output should be interpreted as a target-dependent computational ranking rather than as evidence of a general docking advantage of F-AuNPs across all screened proteins.

Overall, the in silico docking results showed that HPSE and MMP2 were among the highest-ranked protein models for F-AuNPs under the applied computational conditions. However, these findings are strictly predictive and hypothesis-generating. They do not demonstrate direct interaction of F-AuNPs with HPSE, MMP2, or any other protein in HepG2 cells, and they do not establish binding, cellular target engagement, functional involvement, or a mechanistic link between the screened proteins and the observed biological responses. Experimental validation would be required to assess any possible protein-specific involvement.

These computational predictions generate hypotheses regarding possible molecular targets but do not establish binding, target engagement, or a causal link to the observed biological activities, all of which require experimental validation.

## 3. Discussion

The algae-assisted synthesis of AuNPs is both inexpensive and eco-friendly since no dangerous chemicals are used and the synthesis occurs in water under normal conditions. Eco-friendly AuNPs have gained considerable attention due to their wide range of biomedical and technological applications. Algae-based biogenic synthesis of AuNPs is a straightforward, hygienic, affordable, eco-friendly, nontoxic, dependable, and secure method that may be applied in a variety of ways [14]. Examples of AuNP produced with algal extract include *Sargassum wightii* [25], *Laminaria japonica* [26], *Padina tetrastromatica* [23] and *Stoechospermum marginatum* [27].

EDX elemental analysis was performed on the extracted fucoidan-rich fraction to provide supportive elemental evidence for its sulfated polysaccharide nature (Figure 1C). The spectrum showed predominant oxygen (O) and carbon (C) signals, consistent with the carbohydrate backbone of fucoidan, together with a detectable sulfur (S) signal supporting the presence of sulfate-containing groups. Trace inorganic elements, including Na, Mg, K, Ca, and Cl, were also detected, which may originate from the marine algal matrix, residual salts, or the extraction/purification process. The repeated peaks observed for some elements correspond to characteristic X-ray emission lines, mainly Kα and Kβ transitions, rather than separate elemental species or contamination. Although the sulfur signal observed by EDX supports the sulfated nature of the fucoidan-rich fraction, EDX is a semi-quantitative and surface-sensitive technique; therefore, the BaCl_2_–gelatin turbidimetric assay was considered the primary quantitative method for sulfate-content determination, while EDX provided complementary elemental confirmation.

Fucoidans are regarded as some of the most valuable bioactive constituents of brown seaweeds because of their distinctive structural and functional properties. These sulfated heteropolysaccharides have been proposed as promising candidates for the design of biocompatible multifunctional nanoparticle platforms that may enhance photodynamic therapy, support anticancer immune regulation, and help overcome tumor hypoxia [28]. Moreover, fucoidans have been shown to act as natural reducing agents for the environmentally friendly production of antimicrobial silver nanoparticles [29]. In addition, crude seaweed extracts rich in such biomolecules have demonstrated considerable potential for the biosynthesis of both silver nanoparticles (AgNPs) and gold nanoparticles (AuNPs) [30].

In the present study, the sulfate content of the extracted fucoidan-rich fraction was determined using the BaCl_2_–gelatin turbidimetric assay and was found to be 10.08% (*w*/*w*). Sulfate groups represent a defining structural feature of fucoidan and play an important role in determining its biological and physicochemical properties [31]. Previous studies have reported that fucoidans isolated from brown macroalgae typically contain sulfate contents ranging from approximately 5–38%, depending on species and extraction conditions [32]. The presence of sulfur detected in the EDX spectrum further supports the sulfated nature of the extracted polysaccharide [33]. In addition, negatively charged sulfate groups can interact with metal ions and aid in the stabilization and reduction of nanoparticles during green synthesis procedures [34,35].

The bio-organic molecules of fucoidans responsible for the development of gold nanoparticles have been analyzed using FT-IR spectra [36]. The FTIR results in this study made it clear that extracts containing hydroxyl (–OH) functional groups (sugar molecules) are utilized to cap the formation of nanoparticles. According to this study, the synthesis of AuNPs may be related to sugars [37]. The results of previous reports stated that, sugars play a significant part in the synthesis of gold nanoparticles [35]. The FTIR results of the biosynthesis of AuNPs made from *P. tetrastromatica* in the study by Rajeshkumar et al. clearly showed that the extracts containing hydroxyl (–OH) functional groups associated with sugar residues act in capping the nanoparticle formation [34]. Sulfated functional group (C–O–S) was also reported at 838 cm^−1^, confirming the polysaccharide nature of the fucoidan.

The UV–Vis spectrum obtained in the present study exhibited a characteristic surface plasmon resonance (SPR) peak at approximately 544 nm, confirming the successful formation of gold nanoparticles. Similar SPR absorption bands in the range of 520–560 nm have been widely reported for gold nanoparticles synthesized using marine algae extracts. For instance, AuNPs synthesized using the brown alga *P. tetrastromatica* showed a characteristic SPR peak around 540–550 nm [34], confirming nanoparticle formation.

The crystalline structure of the gold nanoparticles produced with fucoidan extract from *P. tetrastromatica* was determined using X-ray diffraction (XRD) patterns. The diffraction pattern exhibited clear Bragg reflections at 2θ values of approximately 38.2°, 44.4°, 64.6°, and 77.5°, corresponding to the (111), (200), (220), and (311) crystallographic planes of face-centered cubic (fcc) gold [34]. The results of this study align with prior research on algae-mediated gold nanoparticles. Furthermore, fucoidan-mediated synthesis of gold nanoparticles from *P. tetrastromatica* has also been reported to produce similar diffraction peaks around 38°, 44°, 64°, and 77°, indicating the formation of crystalline AuNPs with a face-centered cubic structure [34].

To investigate the morphology of the produced AuNPs, TEM analysis was performed. The TEM images indicate that the majority of the nanoparticles are spherical in form, with certain irregularly shaped particles visible. TEM analysis revealed nanoparticle sizes ranging from approximately 10–38 nm with an average particle diameter of 19.44 ± 7.95 nm, confirming the nanoscale dimensions of the synthesized particles. The sulphated polysaccharide of *P. tetrastromatica* is primarily responsible for the formation and stabilization of the nanoparticles. As the solution was not well dispersed, the TEM images showed that the produced AuNPs did not have a uniform size. The homogeneity of the nanoparticles is determined by the preparation technique and reaction conditions. It has been observed that the size of AuNPs is determined by the mixture’s stirring, reaction rate, and reagent incorporation rate [38]. In Rajeshkumar et al. [34], the *Padina tetrastromatica* fucoidan-produced gold nanoparticles (NPs) were discovered to be rounded rectangle, spherical, and pentagon-shaped with a size of 10–70 nm in, confirming the crystallinity with evidence of a circular ring pattern.

Zeta potential provides illumination on colloidal suspensions’ electrostatic stability. While low values suggest poor stability and probable coagulation, high absolute zeta potential negative potential values show strong repulsive forces that inhibit particle aggregation. Colloids with zeta potentials more than 30 mV are often regarded as stable, but those with less than 15 mV typically agglomerate [39]. In the present study, plain AuNPs showed a negative zeta potential of −22.9 ± 5.75 mV and a conductivity value of 1.84 mS cm^−1^, whereas *P. tetrastromatica* F-AuNPs exhibited a slightly more negative zeta potential of −25.4 ± 4.99 mV and a conductivity value of 2.08 mS cm^−1^. This shift toward a more negative surface charge after fucoidan functionalization supports the contribution of anionic sulfate-containing groups within the fucoidan-rich corona to nanoparticle stabilization. The zeta-potential values obtained in this study are consistent with previously reported values for AuNP-based systems. For example, most AuNP samples reported by Ozdemir et al. [40] studies showed ζ-potentials between −20.6 mV and −35.8 mV. In Newehy et al. [33] study, according to zeta potential, the net charge of F-AuNPs from *Sargassum cinereum* was −20.7 mV, with a standard deviation of 3.65 mV and a conductivity of 1.53 mS cm^−1^, whereas the net charge of F-AuNPs from *Turbinaria decurrens* was −24.4 mV, with a standard deviation of 5.23 mV and a conductivity of 2.01 mS cm^−1^. Dynamic light scattering (DLS) analysis showed a hydrodynamic diameter of approximately 99.21 nm with a polydispersity index (PdI) of 0.317, indicating a moderately narrow particle size distribution. The hydrodynamic diameter measured by DLS (~99.21 nm) is larger than the TEM core size (19.4 ± 7.9 nm), which is consistent with the established physical distinction between the two techniques: TEM detects the dry metallic core, while DLS measures the solvated nanoparticle including the fucoidan corona and hydration shell [39]. The estimated corona thickness of approximately 40 nm estimated as half the difference between hydrodynamic and core diameter [(99.21 − 19.4)/2 ≈ 40 nm], consistent with reported hydrodynamic dimensions of high-molecular-weight fucoidan chains (Mw ≈ 1567.6 kDa), which identifies it as having an average hydrodynamic radius (Rh) of 44.5 ± 4.5 nm) of in solution [41]. The moderate polydispersity index (PdI = 0.317) and stable zeta potential (−25.4 ± 4.99 mV) argue against substantial agglomeration as the dominant contributor. Colloidal stability over time and under biologically relevant conditions (PBS, culture and serum-containing media, and varying pH/ionic strength) was not evaluated here. As aggregation, protein-corona formation, or fucoidan desorption may influence biological performance, systematic stability profiling under physiological conditions is identified as an important objective for future work.

To assess the thermal stability of the produced nanoparticles and verify the existence of organic components connected to the nanoparticle surface, thermogravimetric analysis (TGA) was carried out. The thermogram showed a multi-stage weight loss pattern, which is typically attributed to the thermal degradation of organic polysaccharide materials. The initial weight loss is generally associated with the removal of adsorbed moisture, while the subsequent weight loss stages correspond to the decomposition of fucoidan and other organic constituents bound to the nanoparticle surface. Similar to previous studies on brown seaweed fucoidans, thermogravimetric analysis confirmed the thermal decomposition behavior typical of sulfated polysaccharides and supported the structural integrity of the extracted fucoidan [42].

The FRAP assay showed the highest antioxidant activity, with a value of 11.20 ± 0.29 mg TE g^−1^ DW, whereas the DPPH assay exhibited a lower antioxidant capacity of 6.27 ± 0.64 mg TE g^−1^ DW, indicating that the synthesized nanoparticles possessed greater reducing power than free-radical scavenging activity under the tested conditions. This difference may be attributed, at least in part, to the fucoidan-derived surface constituents associated with the nanoparticles, which can influence their redox behavior. In this context, fucoidan from *Fucus vesiculosus* has been reported as a bioactive sulfated polysaccharide with antioxidant potential and has also been used as a reducing and capping agent in the preparation of fucoidan-coated AuNPs for biomedical applications [43,44].

The F-AuNPs were cytotoxic to HepG2 hepatocellular carcinoma cells in a concentration-dependent manner, with an IC_50_ of 138.1 µg mL^−1^. Free fucoidan tested under the same conditions was roughly three times less potent (IC_50_ = 390.2 µg mL^−1^), so conjugation with the gold core measurably improved antiproliferative activity. The lower HepG2 potency of plain AuNPs (IC_50_ 271.2 µg mL^−1^) relative to F-AuNPs (138.1 µg mL^−1^), together with the higher BNL viability and weaker G1 accumulation observed for the plain control, is consistent with a contribution of the fucoidan corona to both antiproliferative activity and normal-cell tolerance, although the underlying mechanism was not directly examined here. The same pattern was reported for fucoidan-based AuNPs from *Turbinaria decurrens* and *Sargassum cinereum*, where the nanoparticle form outperformed free fucoidan in both antioxidant and antiproliferative assays [33]. This enhancement, also seen for fucoidan- and laminarin-derived bioactive compounds from other brown seaweeds, is usually explained by better cellular uptake of the nanoparticle form together with the bioactivity contributed by the sulfated polysaccharide corona [45,46]. The F-AuNPs were far less active against non-tumorigenic BNL hepatocytes. Viability stayed high even at 500 µg mL^−1^, the highest concentration tested, so an IC_50_ could not be determined within this range (IC_50_ > 500 µg mL^−1^). The estimated selectivity index (SI = IC_50_, BNL/IC_50_, HepG2 > 3.62) exceeded the commonly used SI ≥ 3 threshold; however, this value should be interpreted cautiously because the BNL IC_50_ was not reached within the tested range [47]. The low toxicity in BNL cells agrees with what has been reported for sulfated polysaccharide-based nanocarriers, which are generally well tolerated by normal cells [48,49]. Confirmation in additional normal and malignant cell models will be needed before selectivity can be claimed with confidence [50]. Other polysaccharide- and plant-mediated AuNP systems report a wide range of IC_50_ values against HepG2 cells, reflecting differences in capping biomolecules, particle size, and surface chemistry. Broccoli-mediated AuNPs, for instance, gave an IC_50_ of 53.45 µg mL^−1^ against HepG2 cells while keeping HDF viability above 70% [49], and fucoidan-capped AuNPs reported by Rajeshkumar et al. [51] were also active against HepG2 cells, which supports the role of fucoidan as both a reductant and a bioactive capping agent. The IC_50_ obtained here for F-AuNPs sits within the range reported for biogenic AuNPs of similar size and surface composition. The activity likely reflects both the nanoscale properties of the gold core and the sulfated polysaccharide corona, which carries –OSO_3_^−^, –OH, and –COOH groups at the particle surface [33,51]. This interpretation is also consistent with recent reviews highlighting the potential oncology-related applications of fucoidan-based pharmaceutical formulations [52]. However, this study did not directly examine the mechanism of HepG2 cytotoxicity. The observed activity profile is compatible with mechanisms reported for fucoidan- and AuNP-based systems in hepatocellular carcinoma models, where ROS accumulation, glutathione depletion, mitochondrial membrane depolarization, and apoptosis-related signaling have been documented [53]. ROS production, mitochondrial membrane potential, and apoptosis assays will be needed to confirm whether these pathways apply to F-AuNPs and are planned for the next phase of this work.

**Figure 11:**
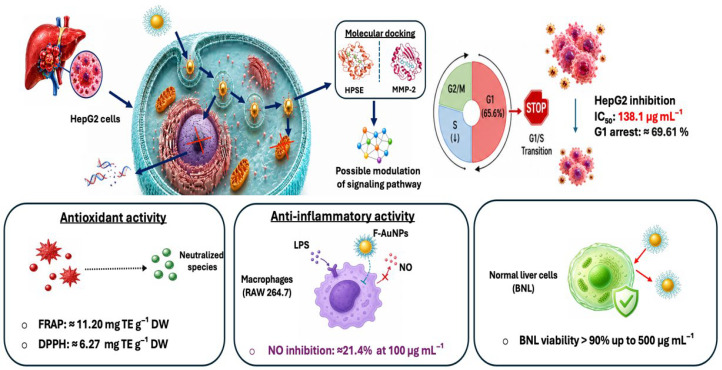
Integrative overview of the in vitro activities and in silico target predictions of *P. tetrastromatica* F-AuNPs. The diagram integrates the observed antioxidant, anti-inflammatory, antiproliferative, and cytocompatibility results with docking-predicted candidate targets (HPSE and MMP-2). The depicted interactions and signaling links are hypothetical and require experimental validation; the figure is intended as an integrative overview rather than a demonstrated mechanism.

The docking analysis identified heparanase (HPSE; heparanase-1) and MMP-2 as the highest-scoring candidate targets of the sulfated fucoidan surface model, with favorable predicted binding in silico that has not been tested experimentally. Both proteins are well-characterized in tumor biology: HPSE is an endo-β-glucuronidase that cleaves heparan sulfate proteoglycans and contributes to extracellular matrix remodeling, tumor progression, and angiogenesis [54,55,56], while MMP-2 is a zinc-dependent endopeptidase that degrades type IV collagen and other matrix components and supports invasion, metastasis, and angiogenesis in cancer [57,58]. Importantly, both targets have been reported to be expressed in HepG2 and other hepatocellular carcinoma cell lines at the mRNA and protein level [59,60] for MMP-2 and [61,62] for HPSE, which supports their biological plausibility as candidates for in silico screening in this model. On this basis, the predicted interactions between the fucoidan-derived corona of F-AuNPs and HPSE or MMP-2 are presented strictly as computational hypotheses, not as evidence of target engagement. The present study did not measure HPSE or MMP-2 expression or activity in the cell lines used, nor did it assess direct binding, enzyme inhibition, or anti-invasive activity; these interactions therefore remain unconfirmed. Reported associations between MMP signaling and natural-compound activity [63] or ROS-related pathways [64] are noted only as broader biological context and were not examined here. Taken together, the docking results provide a hypothesis-generating framework in which the sulfated fucoidan corona of F-AuNPs may engage matrix-remodeling and immune-related proteins. These predictions are independent of, and were not used to explain, the antioxidant, anti-inflammatory, or antiproliferative activities measured here; no causal or mechanistic link between the predicted targets and the observed bioactivities is claimed. Confirmation will require target-specific assays, including expression profiling (qRT-PCR or Western blot) in the cell lines studied, recombinant protein binding, enzyme-inhibition assays, antibody blocking, gene silencing, and nanoparticle uptake analysis. An integrative schematic summarizing the observed in vitro activities of F-AuNPs together with the docking-predicted candidate targets is presented in Figure 11.

Two limitations should be noted. Residual organic solvents in the fucoidan-rich fraction were not quantified. Although ethanol washing and freeze-drying were carried out before nanoparticle synthesis, work intended for pharmaceutical or translational development should include residual solvent analysis using validated chromatographic methods. The MTT assay also did not include a clinically used chemotherapeutic agent as positive control. The IC_50_ values reported here therefore reflect the comparative contribution of the nanoparticle platform relative to free fucoidan rather than a clinical benchmark, and standard reference drugs such as doxorubicin or sorafenib will be incorporated in future validation work.

## 4. Materials and Methods

### 4.1. Collection and Identification of Padina tetrastromatica

The brown alga was manually collected at an approximate depth of one meter from submerged rocky substrates located in front of the National Institute of Oceanography and Fisheries (NIOF), Hurghada, along the Red Sea coast, during the spring season of 2024. Immediately after collection, the algal samples were thoroughly rinsed with ambient seawater to remove adhering sand particles and epiphytic materials. The cleaned specimens were placed in plastic bags containing seawater to prevent dehydration and transported to the laboratory under chilled conditions. Taxonomic identification was carried out by Dr. Mona M. Ismail using standard morphological characteristics in accordance with established classification keys [65]. Species identity was further confirmed using the AlgaeBase online database. Representative photographs of the cleaned algal thalli were taken before drying and extraction to document the raw biological material used in this study.

### 4.2. Solvent-Assisted Extraction and Purification of the Fucoidan-Rich Fraction

The powdered seaweed biomass was subjected to acidic extraction using 0.1 M hydrochloric acid at a solid-to-liquid ratio of 1:10 (*w*/*v*). The extraction mixture was heated at 80–85 °C for 2 h under continuous agitation (200 rpm) in a thermostatically controlled water bath shaker. After the mixture had cooled to room temperature, the extract was filtered to remove insoluble residues. To eliminate alginate and other uronic acid-rich impurities, calcium chloride (CaCl_2_) was added to the filtrate to a final concentration of 1% (*w*/*v*), followed by incubation for 24 h at 4 °C. The mixture was then centrifuged at 3500 rpm for 15 min at 4 °C, and the resulting supernatant was carefully collected.

Fucoidan was precipitated from the supernatant by the addition of chilled ethanol at a ratio of 1:3 (*v*/*v*) and incubated at 4 °C for 24 h. The precipitated fraction was recovered by centrifugation and re-dissolved in distilled water for further purification. Residual proteins were removed by the Sevag deproteinization method using a chloroform: n-butanol mixture (4:1, *v*/*v*). The solution was vigorously shaken and centrifuged to achieve phase separation, after which the upper aqueous phase containing the fucoidan fraction was carefully collected. This step was repeated several times until no visible protein interface remained. The deproteinized solution was then purified by repeated ethanol precipitation. Briefly, chilled absolute ethanol was added to the aqueous phase at a ratio of 1:3 (*v*/*v*), followed by incubation for 24 h at 4 °C. The precipitate was collected by centrifugation, re-dissolved in distilled water, and the precipitation step was repeated twice to further reduce residual salts, organic solvents, and other low-molecular-weight impurities. The final precipitate was washed with absolute ethanol and freeze-dried under reduced pressure for 48 h [66,67].

The extraction and purification process was therefore described as a solvent-assisted procedure rather than a green extraction process. Although the fucoidan-rich fraction was repeatedly washed with ethanol and freeze-dried before nanoparticle synthesis, residual organic solvents were not quantified in the present study.

Using the following formula, the extraction yield was determined as the proportion of the dry weight of the extracted fucoidan-rich fraction to the starting dry weight of the seaweed biomass:

Yield (%) = [Weight of dried fucoidan extract (g) / Initial dry weight of seaweed biomass (g)] × 100

### 4.3. Chemical Analysis of Fucoidan-Rich Fraction

The BaCl_2_–gelatin turbidimetric test was used to measure the sulfate content. In this method, sulfate ions released from the sample react with barium ions under acidic conditions to form a fine barium sulfate suspension, and the resulting turbidity is proportional to the sulfate concentration. Gelatin (0.5 g) was dissolved in 100 mL of deionized water at 60 °C while stirring to produce the BaCl_2_–gelatin reagent, followed by the addition of 0.5 g barium chloride after cooling. Before being used, the reagent was kept at 4 °C.

For analysis, 0.1 g of fucoidan was hydrolyzed with 10 mL of 1–2 M HCl under reflux at 100 °C for 1 h. After cooling, the hydrolysate (0.2 mL) was mixed with 1 mL of BaCl_2_–gelatin reagent, and the mixture was allowed to stand at room temperature for 15 min. The absorbance was then recorded at 360 nm. A calibration curve developed with sodium sulfate as the standard was used to calculate the sulfate content [68].

### 4.4. Preparation of Plain AuNPs and Bio-Assisted Synthesis of Fucoidan-Coated Gold Nanoparticles (F-AuNPs)

#### 4.4.1. Preparation of Plain AuNPs

Plain AuNPs were synthesized as a non-fucoidan nanoparticle control using sodium citrate as a reducing and stabilizing agent, with minor modification from a previously reported citrate-mediated method [69]. Briefly, 50 mL of 1 mM HAuCl_4_·3H_2_O prepared in distilled water was heated to boiling under continuous stirring, followed by rapid addition of 5 mL of 1% (*w*/*v*) trisodium citrate solution. The reaction was continued for 10–15 min until a ruby-red color appeared, confirming AuNP formation. The resulting citrate-stabilized AuNPs were cooled to room temperature and used for comparison with F-AuNPs.

#### 4.4.2. Bio-Assisted Synthesis of Fucoidan-Coated Gold Nanoparticles

At room temperature (25 ± 2 °C), 90 mL of an aqueous chloroauric acid (HAuCl_4_·3H_2_O) solution (1 mM) was continuously magnetically stirred while 10 mL of fucoidan-rich fraction solution (0.1 mg mL^−1^) was added dropwise. Within the first ten minutes of the reaction mixture’s thirty-minute stirring period, a noticeable color shift from light yellow to ruby red was detected, demonstrating the production of gold nanoparticles and the reduction of Au^3+^ ions. Using a UV–Vis spectrophotometer and deionized water as the blank, the reaction mixture’s UV–visible absorption spectra in the 350–700 nm region was recorded at regular intervals to observe the development of AuNP production [70]. In this study, the term bio-assisted synthesis refers specifically to the aqueous nanoparticle formation step, in which the fucoidan-rich fraction served as a reducing and stabilizing agent for fucoidan-functionalized gold nanoparticle (F-AuNP) formation. It does not refer to the preceding solvent-assisted extraction and purification process.

### 4.5. Physicochemical Characterization of Plain AuNPs and F-AuNPs

A UV–Vis spectrophotometer (Shimadzu UV-2101/PC; Shimadzu Corporation, Kyoto, Japan) was used to record the ultraviolet–visible (UV–Vis) absorption spectra of *P. tetrastromatica* F-AuNPs spanning the wavelength range of 200–900 nm to validate nanoparticle production by surface plasmon resonance (SPR). Using Fourier-transform infrared (FTIR) spectroscopy (Model 1430, PerkinElmer, Inc., Waltham, MA, USA) functional groups involved in the reduction and stability of AuNPs have been investigated in the wavenumber range of 400–4000 cm^−1^. An X’Pert Pro diffractometer (PANalytical BV, Almelo, The Netherlands) working over a 2θ range of 20–80° was used to analyze the crystalline structure of F-AuNPs using X-ray diffraction (XRD). Transmission electron microscopy (TEM) was used to evaluate the morphological features, such as particle size and shape (JEM-1400 Plus, JEOL, Akishima, Japan). Dynamic light scattering (DLS) analysis was used to measure hydrodynamic diameter and zeta potential (Nano ZS90, Malvern Instruments Ltd., Malvern, UK). A TGA/DSC 1 STARe System (Mettler Toledo, Greifensee, Switzerland) was used to perform thermogravimetric analysis (TGA). The sample was heated in an atmosphere of nitrogen from 25 to 800 °C at a rate of 10 °C min^−1^. Plain AuNPs were characterized under the same UV–Vis, DLS, TEM, zeta-potential, and TGA conditions and used as a non-fucoidan nanoparticle control for comparison with F-AuNPs.

### 4.6. In Vitro Biological Evaluation of the Fucoidan-Rich Fraction, Plain AuNPs, and F-AuNPs

#### 4.6.1. Antioxidant Activity

##### DPPH (2,2-Diphenyl-1-picrylhydrazyl) Radical Scavenging Assay

DPPH radical scavenging activity of F-AuNPs was assessed using a spectrophotometric method adapted from Sharma and Bhat [71]. In brief, F-AuNP samples were prepared in distilled water at the desired concentrations. Next, in a 96-well microplate, 100 μL of each sample was combined with 100 μL of freshly made 0.1 mM DPPH solution in methanol. The reaction mixtures were incubated at room temperature for 30 min in the dark to allow sufficient interaction between DPPH radicals and antioxidant moieties. After incubation, absorbance was measured at 517 nm using a microplate reader. Trolox was employed as the reference antioxidant standard to construct a calibration curve and express results in terms of Trolox equivalent antioxidant capacity (TEAC), as commonly applied in DPPH assays for diverse antioxidant samples. The following formula was used to determine the percentage of DPPH radical scavenging activity:

Radical scavenging activity (%) = [(C − A) / C] × 100

where A is the absorbance when the tested sample is present and C is the absorbance of the DPPH solution without a sample.

##### Ferric Reducing Antioxidant Power (FRAP) Assay

Reducing capacity of F-AuNPs was assessed using the ferric reducing antioxidant power (FRAP) assay in accordance with the Benzie and Strain theory [72], with minor procedural adaptations. F-AuNPs were prepared in distilled water at a concentration of 12.5 mg mL^−1^. Trolox solutions (25–400 μg mL^−1^) were used to construct a calibration curve. The FRAP working reagent was made fresh by combining 2,4,6-tripyridyl-s-triazine (TPTZ; 10 mM in 40 mM HCl), acetate buffer (300 mM, pH 3.6), and ferric chloride (FeCl_3_; 20 mM) in a volumetric ratio of 10:1:1. In a 96-well microplate (n = 3), 10 μL of each sample was mixed with 190 μL of FRAP reagent for the test. A microplate reader was used to measure the absorbance at 593 nm after a 30-min dark incubation period at room temperature (FLUOstar Omega, BMG LABTECH, Ortenberg, Germany). Trolox equivalents for each gram of dry weight were used to measure antioxidant activity (mg TE g^−1^ DW).

#### 4.6.2. In Vitro Cytotoxicity, Antiproliferative, and Anti-Inflammatory Evaluation of Fucoidan and Fucoidan-Coated Gold Nanoparticles (F-AuNPs)

##### Cell Culture Conditions

Human hepatocellular carcinoma (HepG2) cells, normal mouse liver (BNL) cells, and murine macrophage cells (RAW 264.7) have been purchased from the American Type Culture Collection (ATCC, Rockville, MD, USA). HepG2 cells have been grown in RPMI-1640 medium supplemented with 10% heat-inactivated fetal bovine serum (FBS) and 50 µg mL^−1^ gentamycin. Conversely, BNL and RAW 264.7 cells were maintained in Dulbecco’s Modified Eagle Medium (DMEM) supplemented with 10% heat-inactivated fetal bovine serum (FBS), 100 µg mL^−1^ streptomycin, and 100 U mL^−1^ penicillin. Before being used in experiments, all cell lines were subcultured two to three times a week to maintain logarithmic growth at 37 °C in humid conditions with 5% CO_2_ [73,74].

#### 4.6.3. MTT Cytotoxicity Assay on HepG2 Cells

HepG2 cells were seeded into Corning^®^ 96-well tissue culture plates at a density of 5 × 10^4^ cells per well and allowed to adhere for 24 h. The chemicals examined were subsequently provided to the cells in subsequent concentrations, yielding eight different dosages for each treatment. Vehicle-treated cells receiving culture medium or 0.5% Dimethyl sulfoxide (DMSO) served as controls. Following a 48-h treatment period, the MTT (3-[5-dimethylthiazol-2-yl]-2,5 diphenyl tetrazolium bromide) test was used to assess the cell viability. In summary, 100 µL of phenol red-free RPMI-1640 media was carefully added in place of the treatment medium. Subsequently, 10 µL of MTT solution (12 mM; prepared by dissolving 5 mg mL^−1^ MTT in Phosphate-Buffered Saline (PBS)) was put into every well, including control wells. To allow formazan crystal formation, plates were incubated at 37 °C for four hours. Following incubation, each well’s 85 µL of supernatant was removed, and 50 µL of DMSO was added to dissolve the formazan crystals. Plates were gently mixed and incubated for 10 min at 37 °C. A microplate reader was employed to detect absorbance at 590 nm (SunRise, TECAN, Morrisville, NC, USA). In the present study, comparative MTT analysis was performed among F-AuNPs, plain AuNPs, and the free fucoidan-rich fraction to assess the contribution of the nanoparticle platform and the fucoidan corona to the antiproliferative response. A reference chemotherapeutic drug was not included as a positive control in the present MTT assay; therefore, the reported IC_50_ values should be interpreted as a comparative potency assessment between the nanoparticle and the parent fucoidan rather than as a benchmark against established clinical chemotherapeutic standards. Inclusion of a clinically used reference drug (e.g., doxorubicin or sorafenib) in future studies will allow direct potency benchmarking. The following formula was used to express cell viability as a percentage in relation to untreated control cells:

Cell viability (%) = (ODt / ODc) × 100

The absorbance of treated cells is represented by ODt, while the absorbance of control cells is represented by ODc. GraphPad Prism v9.0.0 (GraphPad Software, San Diego, CA, USA) was used to produce dose–response curves and determine the half-maximal inhibitory concentration (IC_50_) values [74].

#### 4.6.4. Cytotoxicity Assessment of Normal Cells (BNL)—SRB (Sulforhodamine B) Assay

Cell viability of normal mouse liver (BNL) cells following exposure to F-AuNPs was assessed using the sulforhodamine B (SRB) assay. Plain AuNPs were tested in parallel under identical conditions to compare the cytocompatibility of plain and fucoidan-functionalized AuNPs toward normal hepatocytes. Cellular protein content was quantified. Furthermore, in accordance with established procedures, viability was represented as a percentage in relation to untreated control cells [74,75].

#### 4.6.5. Flow Cytometric Analysis of Cell Cycle Arrest

To investigate the antiproliferative mechanism of F-AuNPs, cell-cycle distribution was analyzed by flow cytometry. HepG2 cells (1 × 10^5^) were seeded and treated with plain AuNPs (271.2 µg mL^−1^) or F-AuNPs (138.1 µg mL^−1^), each at its respective HepG2 IC_50_, for 48 h. Untreated HepG2 cells maintained under identical culture conditions without nanoparticle exposure served as the negative control. Following treatment, cells were harvested, washed twice with ice-cold PBS, and fixed in 70% ethanol at −20 °C overnight. Fixed cells were then washed to remove residual ethanol, resuspended in PBS, and stained with propidium iodide (PI; 10 µg mL^−1^) in the presence of RNase A (50 µg mL^−1^) for 30 min at 37 °C in the dark to ensure complete RNA digestion and specific DNA staining. A total of 12,000 events per sample were acquired using a Novocyte™ flow cytometer (ACEA Biosciences, San Diego, CA, USA). Cell populations were first gated using FSC-A on a linear scale and SSC-A on a logarithmic scale to exclude debris and doublets and to select the main viable cell population. DNA content was subsequently determined from PI fluorescence histograms, and the distribution of cells across the G1, S, and G2/M phases was analyzed using NovoExpress software v1.5.0 (ACEA Biosciences) [76].

#### 4.6.6. Anti-Inflammatory Activity in RAW 264.7 Cells

The anti-inflammatory potential of F-AuNPs was evaluated in RAW 264.7 macrophages induced by lipopolysaccharide (LPS) by measuring NO generation using the Griess reaction. Following experimental treatments, culture supernatants were gathered and combined with Griess reagent in an equivalent volume. A microplate reader was used to measure absorbance at 540 nm after a 10-min dark incubation period at room temperature. Nitrite concentrations, as an indicator of NO production, have been calculated using a standard curve for sodium nitrite. A positive anti-inflammatory control was quercetin (30 µM) [77,78].

### 4.7. In Silico Target Prediction and Molecular Docking

An in silico comparative docking workflow was performed to evaluate the predicted interaction profiles of fucoidan-associated and citrate-associated Au models against selected cancer- and extracellular-matrix-related target proteins. The docking analysis was designed as a comparative target-prioritization approach rather than as experimental evidence of direct target engagement or functional mediation in cells.

The ASKCOS-MIT platform was first used for forward reaction prediction [79] to explore whether representative gold-associated structures could be generated from Au and fucoidan- or citrate-related functional groups. Representative fucoidan substructures containing hydroxyl-rich and sulfated fucose moieties were used to generate the fucoidan-associated Au model, whereas citrate-related carboxylate-rich structures were used to generate the citrate-associated Au model. Predicted products with a confidence score of at least 0.5 were considered for further computational analysis. The tested ligand models were therefore defined as a fucoidan-associated Au model, representing fucoidan-functionalized AuNPs, and a citrate-associated Au model, representing citrate-stabilized plain AuNPs. These structures were treated as simplified gold-associated molecular cluster representations and not as experimentally validated models of the complete nanoparticle surface.

Ligand structures were obtained from PubChem when available or generated from representative predicted structures. Ligand geometries were subjected to force-field-based geometry cleanup and energy minimization using the MMFF94 force field implemented in Avogadro software version 1.2.0 [80,81]. For metal-containing or gold-associated cluster models, this minimization step was interpreted only as starting-geometry refinement before docking and not as proof of the true experimental nanoparticle surface structure. The optimized ligands were saved in PDBQT format for docking analysis.

Target proteins were selected based on their relevance to hepatocellular carcinoma biology, extracellular-matrix remodeling, invasion, angiogenesis-associated signaling, and inflammatory processes. SwissTargetPrediction was used to support target identification and prioritization based on the submitted ligand SMILES codes [82]. The selected Homo sapiens targets included heparanase (HPSE; Q9Y251), matrix metalloproteinase-2 (MMP2; P08253), matrix metalloproteinase-1 (MMP1; P03956), protein tyrosine phosphatase receptor type C (PTPRC/CD45; P08575), thymidine phosphorylase (TYMP; P19971), fibroblast growth factor 1 (FGF1; P05230), matrix metalloproteinase-9 (MMP9; P14780), and matrix metalloproteinase-8 (MMP8; P22894). Protein accession identifiers were retrieved from the UniProt database and used to standardize receptor naming. Protein structures were obtained from available structural resources, and AlphaFold v2 was used to predict missing or unresolved structures when experimentally resolved structures were unavailable or incomplete [83].

Protein structures were prepared using AutoDock Tools version 1.5.7 [84]. Polar hydrogens were added, Gasteiger charges were assigned, and water molecules and non-essential heteroatoms were removed before conversion to PDBQT format. Binding-site prediction was performed using the CB-Dock2 server [85], which applies cavity detection and blind docking to identify potential ligand-binding pockets. The top-ranked cavities were selected according to cavity volume and docking score, and the corresponding grid box dimensions were generated automatically.

Molecular docking simulations were performed using QuickVina-2 (QVina2, v2.1) software [86]. The F-AuNPs and plain AuNPs models were docked against the same target panel under identical parameters to allow direct comparison. For each ligand–protein pair, a maximum of 10 binding poses was generated using an energy range of 4 kcal mol^−1^ and an exhaustiveness value of 8. The binding pose with the lowest predicted binding free energy was selected for further analysis. Docking scores were reported as predicted binding affinity values (ΔG, kcal mol^−1^), where more negative values indicate more favorable predicted interactions. Positive docking scores were considered unfavorable computational outcomes and were not interpreted as favorable binding, even when individual contacts were detected.

Docked complexes were visualized and analyzed using PyMOL v2.5 and Discovery Studio Visualizer. Hydrogen bonds, electrostatic interactions, hydrophobic contacts, van der Waals interactions, and metal-associated contacts were inspected for selected complexes. For each ligand–protein combination, docking simulations were repeated in triplicate to assess consistency, and stable docking scores and binding orientations were verified across independent runs. The final docking results were interpreted only as comparative computational predictions and candidate target-prioritization data. They were not used to claim experimentally confirmed binding of F-AuNPs to HPSE, MMP2, or any other target protein.

### 4.8. Statistical Analysis

Statistical analyses were performed using SPSS software version 23.0 and GraphPad Prism. Data are presented as mean ± SD from three independent replicates unless otherwise stated. For comparisons among more than two independent groups, one-way ANOVA followed by Tukey’s post hoc test was used. For grouped datasets involving treatment type and concentration or treatment type and cell-cycle phase, two-way ANOVA followed by Šídák’s multiple-comparison test was applied. IC_50_ values were calculated by nonlinear dose–response regression analysis using GraphPad Prism. Differences were considered statistically significant at *p* < 0.05. Significance levels are indicated as * *p* < 0.05, ** *p* < 0.01, *** *p* < 0.001, and **** *p* < 0.0001.

## 5. Conclusions

The fucoidan-rich fraction extracted from *P. tetrastromatica* showed a sulfate content of 10.08% (*w*/*w*) and an extraction yield of approximately 17.83%, confirming its suitability as a functional biomacromolecular matrix for nanoparticle synthesis. This fraction enabled the formation of F-AuNPs with a characteristic SPR band at approximately 544 nm, a TEM-derived core diameter of 19.44 ± 7.95 nm, a DLS hydrodynamic diameter of 99.21 nm, and a zeta potential of −25.4 mV. The synthesized F-AuNPs also showed measurable antioxidant potential, with FRAP and DPPH values of 11.20 ± 0.29 and 6.27 ± 0.64 mg TE g^−1^ DW, respectively.

The inclusion of plain AuNPs as a non-fucoidan nanoparticle control strengthened the biological interpretation of the study. In HepG2 cells, F-AuNPs exhibited stronger cytotoxic activity than both plain AuNPs and the free fucoidan-rich fraction, with IC_50_ values of 138.1, 271.2, and 390.2 µg mL^−1^, respectively. In normal murine liver BNL cells, F-AuNPs maintained higher cell viability than plain AuNPs across the tested concentration range, indicating improved cytocompatibility associated with fucoidan functionalization. Flow cytometric analysis further confirmed a stronger cell-cycle modulatory effect for F-AuNPs, as reflected by increased G1-phase accumulation compared with both untreated and plain AuNP-treated cells. In addition, F-AuNPs inhibited LPS-induced NO production in RAW 264.7 macrophages by up to 21.42 ± 1.29% at 100 µg mL^−1^.

In silico molecular docking predicted favorable interactions with cancer-related proteins, particularly MMP-2 and HPSE. These predictions are hypothesis-generating only and do not establish target engagement; biochemical and cellular validation, including target expression and binding assays, is required. Overall, the integration of in vitro bioactivity assessment with in silico molecular prediction supports the potential biomedical relevance of *P. tetrastromatica* F-AuNPs, while highlighting target-specific validation as an important direction for future work.

## Data Availability

The original contributions presented in this study are included in the article/Supplementary Materials. Further inquiries can be directed to the corresponding authors.

## Supplementary Materials

The following supporting information can be downloaded at: https://www.mdpi.com/article/10.3390/ph19070976/s1, Figure S1: Calibration curve of sulfate determined by the BaCl_2_–gelatin turbidimetric method using sodium sulfate (Na_2_SO_4_) as the standard. Absorbance was measured at 360 nm; Figure S2: Trolox standard calibration curve used for the determination of antioxidant activity in the FRAP assay. Trolox concentration (µg mL^−1^). Absorbance was measured at 593 nm; Figure S3: Inhibition percentage as a function of Trolox concentration. Trolox concentration (µg ml^−1^). Absorbance was measured at 517 nm; Table S1: Raw TEM-derived particle diameter measurements obtained from 100 individual F-AuNPs and Plain AuNPs using ImageJ/Fiji (2.14.0); Table S2: Comparative molecular docking scores of fucoidan-associated Au and citrate-associated plain Au models against selected cancer- and extracellular-matrix-related target proteins.
