# Supplementary material for: Fucoidan-Mediated Biogenic Gold Nanoparticles from Padina tetrastromatica: In Vitro and In Silico Evaluation of Multifunctional Biological Activities
Source: Pharmaceuticals (Basel). 2026 Jun 23;19(7):976. doi: 10.3390/ph19070976 (PMC13414932; doi:10.3390/ph19070976)
Supplement: Supplementary file 1 [file pharmaceuticals-19-00976-s001.zip › pharmaceuticals-4333085-supplementary.pdf]

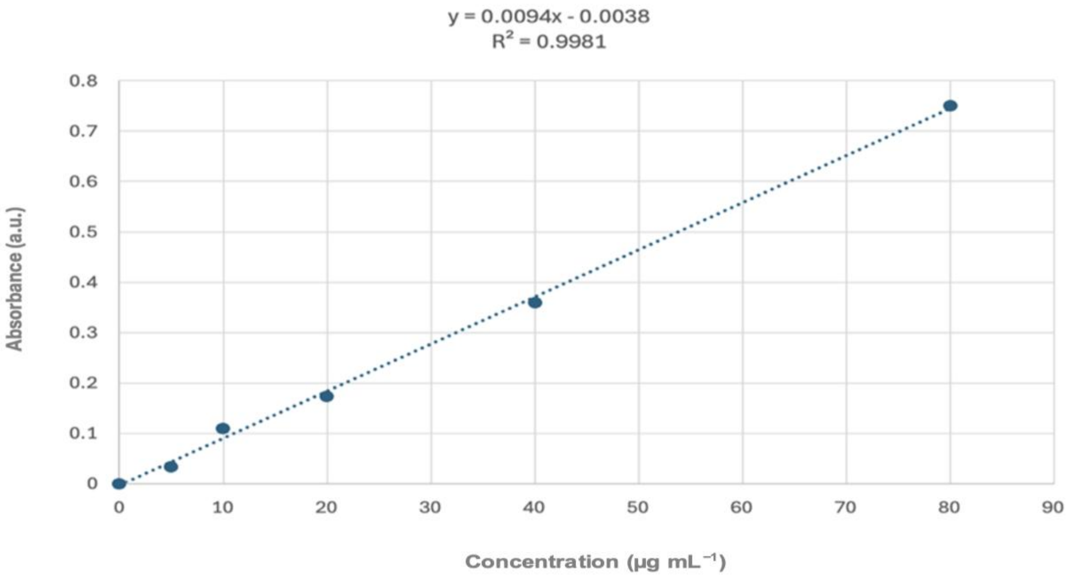

Figure S1. Calibration curve of sulfate determined by the $BaCl_2$–gelatin turbidimetric method using sodium sulfate (Na_2SO_4) as the standard. Absorbance was measured at 360 nm.

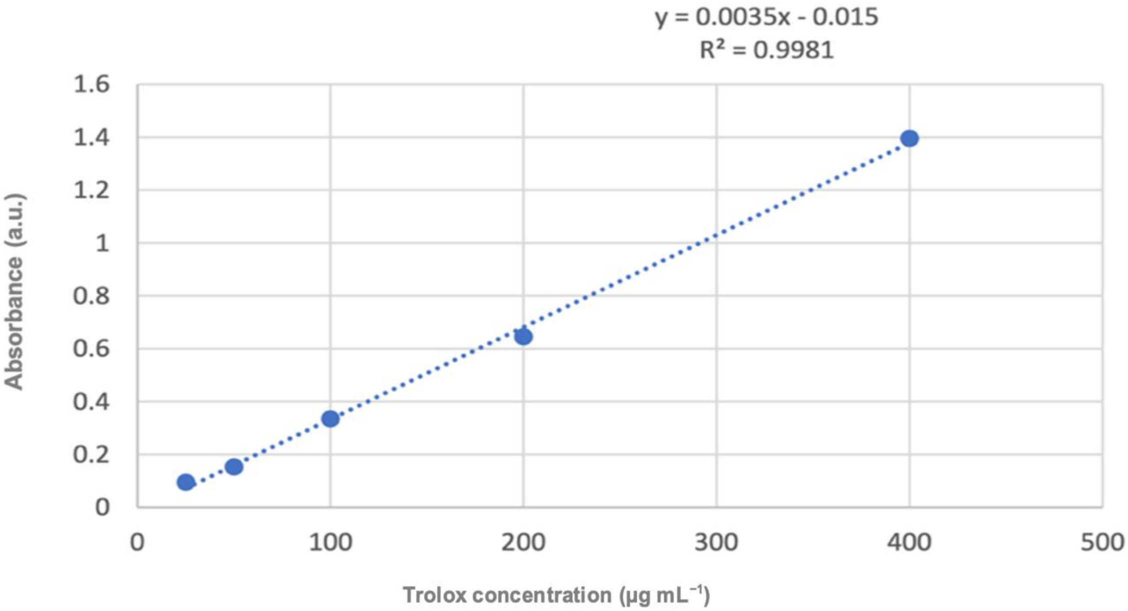

Figure S2. Trolox standard calibration curve used for the determination of antioxidant activity in the FRAP assay.

Trolox concentration (µg mL^{-1}). Absorbance was measured at 593 nm.

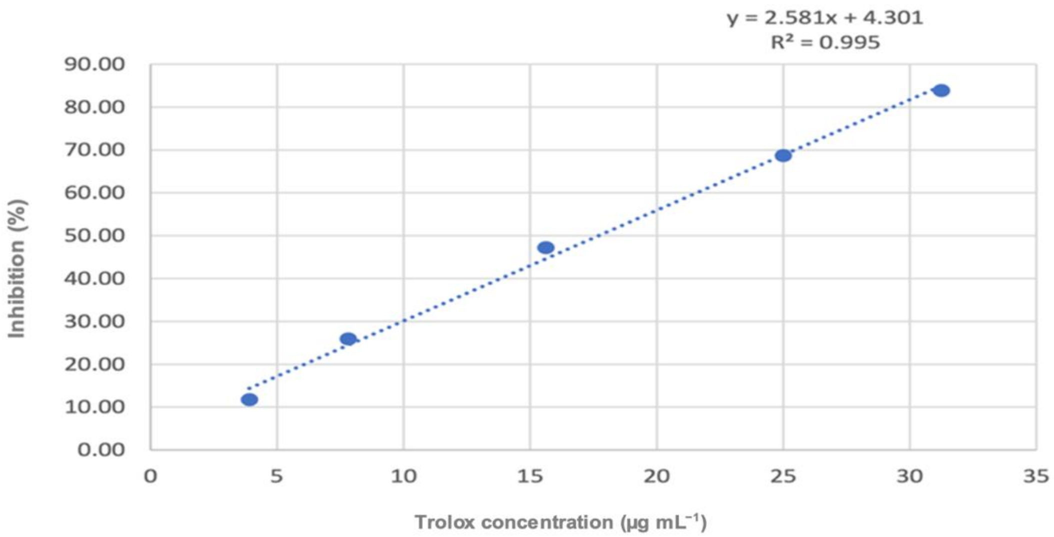

Figure S3. Inhibition percentage as a function of Trolox concentration. Trolox concentration (µg ml^{-1}). Absorbance was measured at 517 nm.

Table S1. Raw TEM-derived particle diameter measurements obtained from 100 individual F-AuNPs and Plain AuNPs using ImageJ/Fiji.

Particle No.	F-AuNPs diameter (nm)	Plain AuNPs diameter (nm)
1	39.333	21.999
2	12.231	17.029
3	27.887	13.152
4	18.114	12.648
5	15.455	12.041
6	11.499	23.536
7	15.535	11.18
8	22.138	15.556
9	8.576	14.865
10	14.200	15.296
11	34.736	12.529
12	17.487	9.486
13	20.124	14.141
14	12.129	9.055
15	27.604	14.141
16	16.083	16.999
17	9.791	14.035
18	28.877	12.206
19	29.105	9.055
20	6.364	14.559
21	12.197	11.045
22	9.000	10
23	32.349	14.317
24	12.204	10.63
25	10.484	12.806
26	39.549	13.038
27	21.789	10
28	20.161	12.648
29	25.013	8.544
30	27.714	14.421
31	9.677	11.661
32	25.013	9.486
33	22.938	12.082
34	18.548	12.999
35	20.161	8.485
36	18.548	9.486
37	31.452	13.341
38	10.969	15.62
39	27.808	8.246
40	26.057	10
41	20.968	11.045
42	24.909	11.045
43	21.774	7.071
44	25.323	16.03
45	17.687	9.434
46	33.919	13.999
47	25.207	13.117
48	13.710	10.811
49	22.027	15.5
50	23.093	16.116
51	11.989	12.613
52	26.007	17.093
53	18.123	11.396
54	20.177	10.545
55	22.710	11.288
56	10.484	14.858
57	19.918	16.241
58	20.812	14.666

59	29.077	9.009
60	9.744	12.993
61	25.000	9.703
62	15.512	12.613
63	14.826	11.85
64	29.839	10.193
65	42.681	16.612
66	24.194	12.613
67	9.439	19.902
68	17.760	12.087
69	11.631	13.514
70	11.631	23.162
71	13.686	10.072
72	9.677	14.527
73	9.677	12.613
74	14.716	10.811
75	13.804	15.315
76	12.545	19.82
77	19.918	14.101
78	19.489	16.216
79	29.433	9.187
80	19.638	22.523
81	9.195	11.712
82	22.407	16.316
83	21.291	15.521
84	34.631	14.997
85	7.661	17.461
86	10.081	12.997
87	26.367	15.649
88	7.651	10.998
89	16.935	12.997
90	16.935	12.039
91	17.705	13.997
92	20.671	12.647
93	24.059	18.024
94	12.310	17.201
95	20.812	15.229
96	12.284	14.997
97	16.309	12.081
98	24.527	14.997
99	23.387	17.996
100	7.608	12.997

Table S2. Comparative molecular docking scores of fucoidan-associated Au and citrate-associated plain Au models against selected cancer- and extracellular-matrix-related target proteins.

Target protein	UniProt ID	F-AuNP model ΔG (kcal mol^{-1})	Plain AuNP model ΔG (kcal mol^{-1})	ΔΔG (F-AuNP − Plain AuNP)
**HPSE**	Q9Y251	−13.87	−9.03	−4.84
**MMP2**	P08253	−13.42	−8.91	−4.51
**MMP1**	P03956	−13.15	−9.95	−3.20
**PTPRC**	P08575	−12.60	−8.03	−4.57
**TYMP**	P19971	−12.15	−11.57	−0.58
**FGF1**	P05230	−9.76	−8.44	−1.32
**MMP9**	P14780	1.84	−11.96	13.80
**MMP8**	P22894	1.41	−11.94	13.35

Values are expressed as predicted docking scores in kcal mol^{-1}. More negative values indicate more favorable predicted interactions. ΔΔG was calculated as the docking score of the F-AuNP model minus the docking score of the plain AuNP model. Negative ΔΔG values indicate more favorable predicted docking for the F-AuNP model, whereas positive ΔΔG values indicate more favorable predicted docking for the plain AuNP model. These data are intended for comparative computational target prioritization only and do not confirm direct target engagement or functional mediation in cells.
